# Network Anomaly Intrusion Detection Based on Deep Learning Approach

**DOI:** 10.3390/s23042171

**Published:** 2023-02-15

**Authors:** Yung-Chung Wang, Yi-Chun Houng, Han-Xuan Chen, Shu-Ming Tseng

**Affiliations:** 1 Department of Electrical Engineering, National Taipei University of Technology, Taipei 106, Taiwan; 2 Department of Electronic Engineering, National Taipei University of Technology, Taipei 106, Taiwan

**Keywords:** deep learning, network intrusion detection, data processing

## Abstract

The prevalence of internet usage leads to diverse internet traffic, which may contain information about various types of internet attacks. In recent years, many researchers have applied deep learning technology to intrusion detection systems and obtained fairly strong recognition results. However, most experiments have used old datasets, so they could not reflect the latest attack information. In this paper, a current state of the CSE-CIC-IDS2018 dataset and standard evaluation metrics has been employed to evaluate the proposed mechanism. After preprocessing the dataset, six models—deep neural network (DNN), convolutional neural network (CNN), recurrent neural network (RNN), long short-term memory (LSTM), CNN + RNN and CNN + LSTM—were constructed to judge whether network traffic comprised a malicious attack. In addition, multi-classification experiments were conducted to sort traffic into benign traffic and six categories of malicious attacks: BruteForce, Denial-of-service (DoS), Web Attacks, Infiltration, Botnet, and Distributed denial-of-service (DDoS). Each model showed a high accuracy in various experiments, and their multi-class classification accuracy were above 98%. Compared with the intrusion detection system (IDS) of other papers, the proposed model effectively improves the detection performance. Moreover, the inference time for the combinations of CNN + RNN and CNN + LSTM is longer than that of the individual DNN, RNN and CNN. Therefore, the DNN, RNN and CNN are better than CNN + RNN and CNN + LSTM for considering the implementation of the algorithm in the IDS device.

## 1. Introduction

Due to the vigorous development of technologies such as the Internet of Things (IoT), cloud computing, and 5G communication, there are many applications that make the prevalence of the Internet. The popularization of Internet has occurred in parallel with an increasing number of hackers’ attack strategies. According to Acronis’ Cyber Threat Report [1], the main attack methods in 2021 were phishing, ransomware, and cryptocurrency. These attacks penetrate networks through system vulnerabilities and send large amounts of malicious information by email. The cryptocurrencies, in particular, have been attracting hackers who use malicious software to steal digital assets due to the high number of investors in recent years [2]. In the future, there will be more attacks on automated transactions. Therefore, strengthening network security to prevent disasters in the case of the simultaneous development of the digital world and network attacks has become an important issue.

There are many ways to prevent hacker intrusion. In addition to a firewall as the first line of defense, the second line of defense is an intrusion detection system, which is used to monitor network traffic for abnormal behavior. An IDS collects a large amount of malicious attack data in advance and compares their behavior patterns with the attack characteristics of the database to determine whether they comprise intrusion information to conduct effective defense against new ransomware. Deep learning involves a neural network with a multi-level architecture, which is different from a machine learning network in that it can learn and process features by itself and then generate changes in feature values from the architecture. The deep learning of automatic feature processing engineering is the most efficient method for dealing with the rapid rise of big data, and appropriate combinations of neurons and layers should be designed to extract important features and make judgments for large amounts of data. In [3,4], the author listed many papers on the application of deep learning in network attack detection. Therefore, it is suitable to use deep learning to implement IDS.

Whether deep learning is successfully applied to IDS, the network intrusion detection datasets of the training model is very important. Accordingly, research has been conducted on publicly available network intrusion detection datasets; the most commonly researched datasets have been KDD Cup 1999 (KDD99) and NSL-KDD [5]. The network traffic of these two datasets have been sufficient to detect intrusion-spreading viruses, but today’s attack methods have diversified so these datasets are outdated and unreliable [6]. The CSE-CIC-IDS2018 dataset is derived from real network traffic data, and it can be applied to actual network detection capabilities through deep learning methods [7,8]. The lack of data volume and feature types has led to an inability to prevent current damage trends, so we used the latest network intrusion detection CSE-CIC-IDS2018 dataset for our experiments. That fact allows us to evaluate the capability of deep learning methods to work in real network. 

In this paper, we used the CSE-CIC-IDS2018 dataset for intrusion detection experiments. Because a large amount of data may cover repeated values, we also focused on data processing. In addition, we applied DNN, CNN, RNN, LSTM, CNN + RNN and CNN + LSTM models to detect network attacks. Finally, binary classification and multi-class classification tasks can be performed to judge whether traffic is a malicious attack. The main contributions of this paper are summarized as follows.

1. This paper uses NVIDIA GPU to accelerate the training procedure. We used the complete CSE-CIC-IDS2018 dataset to reflect the current network traffic conditions in our experiments, with a focus on data preprocessing, to provide comprehensive test results. We adopted the DNN, CNN, RNN, LSTM, CNN + RNN and CNN + LSTM models to handle binary and multi-class classification tasks. When using the proposed appropriate data preprocessing methods and systematically tune hyperparameters of all six models, the accuracy of all models was found to be above 98%. Compared with the IDS of other papers, the proposed model effectively improves the detection performance.
2. Along with the empirical demonstration, the inference time for the combinations of CNN + RNN and CNN + LSTM is longer than that of the individual DNN, RNN and CNN. When considering the implementation of the algorithm in the IDS device, we conclude that individual DNN, RNN and CNN are better than CNN + RNN and CNN + LSTM.

The remainder of this paper is organized as follows. Related work is discussed in Section 2. In Section 3, we illustrate the methodology. In Section 4, we present experimental results, and Section 5 presents the conclusions.

## 2. Related Work

Under the current trends of internet popularization and the continuous growth of hacking, many researchers have applied deep learning methods to the field of network security to more effectively detect new and complex types of network attacks. In deep learning networks, multiple layers of nonlinear transformations automatically process information. If large amounts of data are considered by a deep learning method, the neural network learning characteristics of the multi-layer structure can be effectively utilized to obtain more accurate results. The existing deep learning approaches are introduced below and the summary of them is shown in Table 1.

In Ref. [9], Xiao et al. applied CNN for IDS to pull out the features of dimensionality reduction data. The authors of Ref. [10] proposed a wireless network intrusion detection method based on an improved CNN. In Ref. [11], Lin et al. proposed an LSTM + AM (attention mechanism) model to enhance the recognition ability of the network. The LSTM method has memory characteristics that can grasp historical network traffic. In a hierarchical neural network structure, LSTM can effectively combine current data and previously learned features to achieve better classification results. The essence of deep learning is to imitate the operation mode of the human neural network. The structure of LSTM is similar to the memory ability of the human brain, and AM is similar to the attention mechanism of the brain. 

In Ref. [12], Karatas et al. used the CSE-CIC-IDS2018 dataset and proposed six machine learning algorithms, namely, adaptive boosting (AdaBoost), DT, random forest (RF), KNN, gradient boosting (GB) and linear discriminant analysis (LDA). In order to solve the problem of an unbalanced number of attack types, the synthetic minority oversampling technique (SMOTE) can be used to synthesize new samples to improve the detection efficiency of a few samples. In Ref. [5], Ferrag et al. used the Bot-IoT and CSE-CIC-IDS2018 datasets to analyze seven deep learning methods, namely, DNN, RNN, CNN, restricted Boltzmann machine (RBM), deep belief network (DBN), deep Boltzmann machine (DBM) and deep autoencoder (DAE). In Ref. [13], Hu et al. employed the CNN and the adaptive synthetic sampling (ADASYN) to strengthen IDS. In Ref. [14], Jiang et al. combined hybrid sampling with CNN and bi-directional long short-term memory (BiLSTM) for network intrusion detection. In Ref. [15], Jiang et al. applied LSTM-RNN to implement a multi-channel attack detection system. The author proposed an end-to-end framework that integrated data preprocessing, feature extraction and training, as well as detection. In Ref. [16], the author proposes a security protection platform for the control plane of a software defined network. They employ LSTM and CNN to implement an efficient and timely IDS.

In Ref. [17], Kim et al. used the KDD99 and CSE-CIC-IDS2018 datasets, which contain a variety of attack types, but the authors only focused on denial of service (DoS) attacks, which were comparable in number to those of other attack samples and contained enough samples to train and test a proposed CNN architecture. The convolution layer in its structure is conducive to extracting important features from a large amount of data. For this reason, the authors converted the feature vector of the data into an image so that their CNN could better display its image recognition capabilities. In Ref. [6], Mezina et al. used the KDD99 and CSE-CIC-IDS2018 datasets and applied a U-Net and temporal convolutional network (TCN) to identify network attacks. They combined a TCN with LSTM to achieve a higher accuracy; the TCN mainly solved time series data by adding dilated convolution, which was able to increase the scope of convolution. In Ref. [18], Imrana et al. proposed a BiDLSTM to implement IDS to detect User-to-Root (U2R) and Remote-to-Local (R2L) attacks. The proposed BiDLSTM achieved a higher accuracy than the LSTM. In Ref. [19], the author applied principal component analysis and a mutual information technique to reduce dimension and select features of the dataset. Then, they employed LSTM to implement detecting attacks mechanism.

The authors of Ref. [20] applied CNN and CNN-LSTM to secure the autonomous vehicle from intrusions. They trained and evaluated methods on a real automatic vehicle network dataset, including spoofing, flood, replaying attacks, and benign packets. In Ref. [21], Tang et al. proposed a deep stacking network (DSN) model which combined the predictions of multiple classifiers to improve the classification accuracy of IDS.

## 3. Methodology

**Figure 1:**
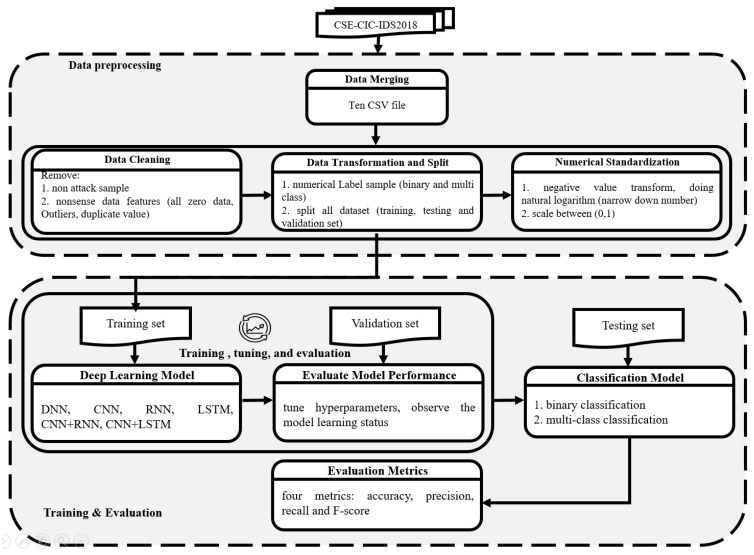
Methodology diagram.

The methodology of our network intrusion detection model is shown in Figure 1. The diagram is divided into two parts. The first is the data preprocessing area, and the second is the training and evaluation area. Before model training, it was necessary to further understand the network traffic of the experimental dataset and the characteristics of the data.

### 3.1. CSE-CIC-IDS2018 Dataset

This paper used the CSE-CIC-IDS2018 dataset [22] for experimental evaluation. The CSE-CIC-IDS2018 dataset was established by the Canadian government’s Communications Security Establishment (CSE) [23] and the Canadian Institute for Cybersecurity (CIC) [24] in cooperation with Amazon Web Services (AWS) [25]. It is the latest, most comprehensive and large-scale dataset among the publicly available intrusion detection datasets on the internet. 

The CSE-CIC-IDS2018 dataset is a ten-day dataset comprising data collected through the network topology of authentic network attacks, and it stores benign traffic and attack traffic in the CSV file format. The dataset has a total of 10 files with total size of 6.41 GB [22]. The total number of datasets in the CSE-CIC-IDS2018 is 16,233,002 [7]. Due to this huge number and the presence of redundant data, the official dataset did not provide divided training and testing samples. So far, studies have presented inconsistent results regarding the total amount of data obtained and the data processing methods used. For example, the authors of [26] randomly selected 40,000 benign data (the total number of benign traffic data was 13,484,708) and 20,000 attack data to conduct experiments. The authors of [6] used nine of the ten files for their experiments. In this study, we used all datasets for experimental evaluation. The dataset records a series of packets, including 83 data characteristics such as duration, number of packets and number of bytes.

In the CSE-CIC-IDS2018 dataset, the last item of each sample data is a label that represents whether the network traffic is of the benign or attack types. The attack type is divided into six categories for a total of 14 kinds of attacks, as shown in Table 2.

### 3.2. Data Preprocessing

Since the total number of datasets was large, they could have contained features or outliers that would not have been helpful for training. If there was no proper preprocessing, the trained model would not have been able to identify various intrusion attacks. To this end, this study was focused on data preprocessing, including data merging, data cleaning, data transformation and split, and numerical standardization.

For feature extraction in deep learning, the coefficient of the number of layers must be set, and the larger the number of layers, the larger the processing scale of a feature. According to Anaconda’s 2020 engineering survey of data scientists investing in deep learning [27], in the deep learning field, nearly 50% of time is spent on feature engineering, including data cleaning, data conversion and text cleaning. In data analysis, there is a famous saying, “Garbage In, Garbage Out”, which means that the input error or meaningless data are of the same nature as the output data. Therefore, before model training, the preprocessing of data must be conducted. Unprocessed raw datasets usually come from diverse sources, which means that the data may have many non-numeric formats that cannot be read by computers, as well as missing values and noise. After these problems are resolved, high-quality data are obtained and then input into a model. Training is then conducted to achieve results with a low number of false positives and the best possible accuracy.

The data preprocessing in this study comprised data merging, data cleaning (non-attack data, feature removal, outliers and duplicate values), data transformation, the split of training and test sets, and numerical standardization. After synthesizing the previous preprocessing, the total number of datasets was reduced from 16,233,002 to 10,114,753. Each of the preprocessing steps are explained in detail in the following sections.

#### 3.2.1. Data Merging

The CSE-CIC-IDS2018 dataset has ten CSV files, and each file has benign traffic and different attack traffic. The attack data in file No. 1 is brute force attack; No. 2 and 3 are denial of service attacks; No. 4 and 5 are website attack; No. 6 and 7 are penetration attacks; No. 8 is a botnet attack; No. 9 and 10 are distributed denial-of-service attacks. Ten files must be combined into one file before loading the data for processing.

#### 3.2.2. Data Cleaning

It is necessary to process the string data and errors that are not helpful for the training process or cannot be processed through numerical operations. The objective of processing in this study was to delete meaningless data features, outliers and repetitive data. Before entering the stage of deleting meaningless data features, the non-attack data labels, which are not officially described as benign or attack traffic, were first deleted. Next, the meaningless data features were deleted. First, we found that the six features of Timestamp, Flow ID, Src IP, Src Port, Dst IP and Dst Port in the dataset had no effect on the attack classification of network traffic, so they were removed. If considered data features all have values of 0, there would be no discrimination during training. Here, it was found that the values of Bwd PSH Flags, Bwd URG Flags, Fwd Byts/b Avg, Fwd Pkts/b Avg, Fwd Blk Rate Avg, Bwd Byts/b Avg, Bwd Pkts/b Avg and Bwd Blk Rate in the eight features had values of 0, so they were deleted.

Our second data cleaning stage was the processing of outliers. There were two types of outliers in the dataset: Not a Number (NaN) and Infinity (Inf). There are methods to deal with NaN, such as average value, mode filling or deletion. It was found that there were abnormal values in the Flow Byts/s and Flow Pkts/s characteristics. We used the mode filling processing method instead of average filling to avoid changing the original data values, as the averaged values may have been affected by other outliers. 

The last step was to delete the repetitive data. The number of deletions in each stage and the total number of datasets after cleaning are shown in Table 3.

#### 3.2.3. Data Transformation and Split

**Figure 2:**
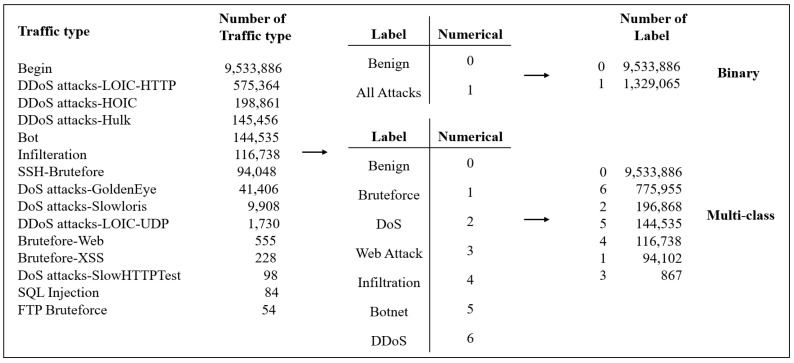
Benign and offensive labels, digital representation, and binary and multi-class classification.

Figure 2 shows the benign and attack labels of the dataset, which are all in the text format. Since a computer cannot understand non-numeric data, they must be converted into the numerical format so that a model can read them for training. In this paper, a classification index was adopted. Processing was used to classify the attack data into binary and multi-class categories. The binary classification assigned each label an integer of 0 or 1 for the benign and attack samples, respectively. The multi-class classification benign samples were assigned a value of 0, and the remaining six attacks were classified as Bruteforce (1), DoS (2), Web Attack (3), Infiltration (4), Botnet (5) and DDoS (6) digital representation. Finally, we used one-hot encoding conversion. Because DDoS is composed of DDoS attacks-LOIC-HTTP, DDoS attack-HOIC and DDoS attack-LOIC-UDP, the number of label 6 is 575,363 + 198,861 + 1730 (775,955) in Figure 2.

Because the dataset does not provide training and test samples, in this paper, we adopted the holdout method for split processing. This method is used to divide datasets into a training-validation set and testing sets according to a set ratio; the division ratio has no uniform requirement, as it is completely set by experience. In this experiment, 80% and 20% of the dataset were set as the training-validation set and testing data, respectively. This division allowed the model to have a generalization effect. Moreover, 80% and 20% of the training-validation dataset were set as the training set and validation set. The number of categories and the proportions of the experimental training-validation data and testing data are shown in Table 4 and Table 5. 

#### 3.2.4. Numerical Normalization

The data range of each feature in the original dataset is different. We used the standardization method to change the mean of the original data to 0 and the standard deviation (SD) to 1 to scale each feature data, ensure that the data conformed to a normal distribution, and improve the convergence speed and accuracy of the model. The equation is shown in (1), where *x* is the original value to be standardized, *μ* is the average value of the feature, and *σ* is the standard deviation of the feature.

(1) $$z=\frac{x-\mu }{\sigma }$$

After the data were standardized, an interval between the values still existed. Therefore, before using the natural logarithmic transformation to narrow the numerical range, the eight features with negative values were transformed to solve the problem of negative numbers without the use of a logarithm. Finally, if the data contained 0 values that could be logarithmically solved, we applied $\log_{e}\left(1+x\right)$, where *x* is the original value to be converted that cannot be less than 0.

### 3.3. Deep Learning Models

This paper used DNN, CNN, RNN, LSTM, CNN + RNN and CNN + LSTM for experiments. The two combined CNN + RNN and CNN + LSTM models used a CNN because we hoped to use their feature extraction characteristics to combine the best data feature extraction capabilities with time series properties to achieve efficient classification results.

In addition to the input and output layers, a deep learning model contains a neural network with several hidden layers. However, deep learning is by no means completed by stacking multiple layers of neural networks. Sometimes, a network structure with a small number of layers combined with dropout and batch normalization can also have good results. At present, no research has defined any formula to calculate an optimal number of neural network layers and neurons. Too many neurons may lead to overfitting, which means that the training set data differ from the number of neurons. If a network is too large, it cannot cope with the learning process; if a network is too small, it will cause underfitting, which means that the learning degree is insufficient. When designing the neural network architecture, we continuously tested the results with various combinations and finally chose the appropriate number of neurons.

In this paper, we tested various combinations of specific neural network node numbers, learning rates and excitation functions. The number of nodes in a neural network is proportional to the number of parameters. The learning rate directly affects the weight update during the operation of the back propagation method, which consequently affects the convergence of the model. The learning rate affects the learning speed and the time required for training, which should be determined according to the size of the considered dataset. In this paper, we set the learning rate range from 0.01 to 0.5 for our experiments, and we designed various combinations of neural networks in shallow to deep learning experiments to find suitable models and architectures, as shown in Table 6. The hidden layer was set to comprise 1 to 5 layers. The total numbers of neurons in the hidden layers were set to 256, 512 and 768.

#### 3.3.1. DNN

Table 7 shows the DNN architecture used in this paper, which consisted of five hidden layers. Layers 0~1 are the input layers and the hidden layer. Layers 3~4, 6~7, 9~10 and 12~13 are all hidden layers. Layers 16~17 are output layers.

The number of the layer parameters of the DNN was calculated as (number of input features × number of nodes) + deviation value. The number of features after data processing was 70; that is, the first 69 items were data features and the last item was a label. The number of first layer parameters was 4480 (shown in Table 6), and it was calculated as 69 × 64 + 64. The function of the deviation value was to excite a neuron and make the next neuron more conducive to receiving data, so the value was based on the number of nodes. Furthermore, the remaining layers were calculated in the same way.

In the training phase, overfitting often occurs and results in a reduction in the generalization ability of a model. Therefore, in this experiment, batch normalization (BN) [28] and dropout layers were added between each hidden layer. BN can speed up the training process and prevent overfitting. Dropout randomly deletes neurons in each layer at a ratio. Both are effective in preventing neurons from over-relying on certain local features. Our purpose for using BN was to change the mean value of original data to a normal distribution with a standard deviation of 0 and a standard deviation of 1. BN was normalized for each batch in the training phase, and then we added two elements that controlled the size of the value, namely, scaling and offset. Through the normalization process during training, a value with a more even distribution could be obtained, which further improved the convergence speed of the model. BN has four calculation parameters, namely, mean, standard deviation, scaling and offset control, and these four parameters were applied to all data. Table 6 shows the parameter amount of the BN layer, which was calculated as 64 times 4 times the number of parameters. The number of the dropout layer parameters in Table 6 is 0, because the dropout layer’s role was only to drop neurons. The number of parameters of the DNN model was shown in Table 8.

#### 3.3.2. CNN

Table 9 shows the CNN architecture used in this paper. The architecture consisted of five convolutional layers. Layers 0~1 comprised the input layer and the first convolutional layer. Layers 2~3, 4~5, 6~7 and 8~9 were all convolutional layers, and layers 13~14 were output layers.

In operation of the convolutional layer, the filters and the kernel are used to calculate the input data according to stride movement. In Table 8, the number of filters in the convolutional layer is shown to be 32. The kernel size is the window size of the convolution kernel, and its value was set to 2 × 1. The number of the layer parameters was calculated as the number of filters × (filter height × filter width × input channel) + deviation value. The number of first layer parameters was 96 (shown in Table 8), and it was calculated as 32 × (2 × 1 × 1) + 32. The remaining layer to the output layers were calculated in the same way. In the CNN architecture, the BN and dropout layers were added before the output layer because the maximum pooling layer of 1 and 2 could effectively prevent overfitting. The feature map after convolution could be extracted, focusing on important data and reducing meaningless noise. Therefore, the output dimension of each maximum pooling layer was increased. The output dimension of the convolutional layer was half of the output dimension, and the reduction in the number of parameters also retained important characteristics. The number of parameters of the CNN model was shown in Table 10.

#### 3.3.3. RNN

Table 11 shows the RNN architecture used in this paper, which consisted of five recurrent layers. Layers 0~1 were the input layer and the first recurrent layer. Layers 3~4, 6~7, 9~10 and 12~13 were all recurrent layers. Layers 15~16 were output layers.

The operation mode of the RNN is different from that of a DNN. It has an inner loop structure. The number of the layer parameters was calculated as (number of input features × number of nodes) + (nodes number × number of nodes) + bias value. The number of the first layer parameters was 8576 (shown in Table 10), and it was calculated as (69 × 64) + (64 × 64) + 64. In the second layer, the number of input features was changed to 64 of the output shape of the previous layer, so the number of parameters was 8256, and it was calculated as (64 × 64) + (64 × 64) + 64. The remaining hidden layers were calculated in the same way. In the RNN architecture, the placement of the BN and dropout layers was set as the same as that of the DNN in order to effectively avoid overfitting. The number of parameters of the RNN model was shown in Table 12.

#### 3.3.4. LSTM

Table 13 shows the LSTM architecture used in this paper, which consisted of five LSTM layers. Layers 0~1 were the input layer and the first LSTM layer. Layers 3~4, 6~7, 9~10 and 12~13 were all recurrent layers. Layers 15–16 were output layers.

The LSTM structure used forgetting gates, input gates, update gates and output gates to determine whether the data were added to the memory and to improve the problem of lack of long-term memory. These four control gates had four sets of parameters. The number of parameters of the RNN was calculated as 4 × (number of input features × number of nodes) + (number of nodes × number of nodes) + deviation value. The number of first layer parameters was 34,304 (shown in Table 12), and it was calculated as 4 × [(69 × 64) + (64 × 64) + 64]. The input feature number of second layer was changed to 64 of the output shape of the previous layer. The number of second layer parameters was 33,024, and was calculated as 4 × (64 × 64) + (64 × 64) + 64. The hidden layers were calculated in the same way. In the LSTM architecture, the placement of BN and dropout layers was consistent with the DNN and RNN. The number of parameters of the RNN model was shown in Table 14.

#### 3.3.5. CNN + RNN

RNN have time-series characteristics that aid the detection of benign and attack traffic sequence. Combining RNN with the feature extraction characteristics of CNN can effectively improve the identification ability, which proves that the hybrid model does have a higher accuracy of network traffic classification. Table 14 shows the CNN + RNN architecture used in this paper, which consisted of three convolutional layers and five recurrent layers. Layers 0~1 were the input layer and the first convolutional layer. Layers 2~3 and 4~5 were convolutional layers, and layers 8~9, 11~12, 14~15, 17~18 and 20~21 were all recurrent layers. Layers 23~24 were output layers. The number of parameters of CNN + RNN was calculated as filter number × (filter height × filter width × input channel) + deviation value and (input feature number × node number) + (node number × node number) + deviation value. The number of parameters of the first convolutional layer was 32 × (2 × 1 × 1) + 32 = 96. The number of parameters of the first recurrent layer was (128 × 64) + (64 × 64) + 64 = 12,352, as shown in Table 15. The number of parameters of the RNN model was shown in Table 16.

#### 3.3.6. CNN + LSTM

LSTM networks have time-series characteristics that aid the detection of benign and attack traffic sequence. Combining LSTM with the feature extraction characteristics of CNN can effectively improve the identification ability, which proves that the hybrid model does have a higher accuracy of network traffic classification. Table 17 shows the CNN + LSTM architecture used in this paper, which consisted of three convolutional layers and five LST layers. Layers 0~1 were the input layer and the first convolutional layer. Layers 2~3 and 4~5 were convolutional layers, and layers 8~9, 11~12, 14~15, 17~18 and 20~21 were LSTM layers. Layers 23~24 were output layers. The number of parameters of CNN + LSTM was calculated as filter number× (filter height × filter width × input channel) + deviation value and 4 × (number of input features × number of nodes) + (number of nodes × number of nodes) + deviation value. The number of parameters of the first LSTM layer was 4 × (128 × 64) + (64 × 64) + 64 = 49,408. The number of parameters of the RNN model was shown in Table 18.

### 3.4. Evaluation Metrics

This paper used four elements to judge the number of correct and misjudged results predicted by the experimental model. This involved four elements, namely, (1) true positive (TP), which is the number of correctly classified benign samples; (2) false positive (FP), which is the number of false positives that will attack the number of samples predicted as benign samples; (3) true negative (TN), which is the number of correctly classified attack samples; and (4) false negative (FN), which is the number of false positive samples predicted as attack samples. With these four elements, four evaluation indicators could be calculated to evaluate the performance of the experimental model [29] in terms of accuracy, precision, recall and F1-score. Accuracy represents the ratio of correct classifications of each sample. Precision represents the correctness of the prediction in the case of benign samples. Recall indicates the correct rate in the case of a benign sample. F1-score represents the harmonic mean of the precision and the recall, which is an indicator of the performance of the classification model. The equations are (2)~(5).

(2) $$Accurcay=\frac{TP+TN}{TP+TN+FP+FN}$$

(3) $$Precision=\frac{TP}{TP+FP}$$

(4) $$Recall=\frac{TP}{TP+FN}$$

(5) $$F1\text{-}Score=2\times \frac{Precision\times Recall}{Precision+Recall}$$

## 4. Experimental Results and Analysis

### 4.1. Experimental Environment

The experimental environment used the VCP-AI computing platform of Taipei Tech, along with GPU computing resources to speed up neural network-like processing. The detailed specifications and training speed of the environment are shown in Table 19. The experimental development language was Python. We used the glob tool [30] to obtain a list in a serial manner for subsequent data merging. When the amount of studied data is large, Pandas [31], NumPy [32] and scikit-learn tools can be used to perform efficient data processing and analysis.

### 4.2. Results and Analysis

In this section, the multi-class and binary classification experimental results of six neural network models—DNN, CNN, RNN, LSTM, CNN + RNN and CNN + LSTM—are shown and discussed.

#### 4.2.1. Evaluation of Multi-Class Classification

The best multi-class classification accuracy of the DNN was 98.83%. The best multi-class classification accuracy of the CNN was 98.83%. The multi-class classification accuracy of the RNN and LSTM were 98.80% and 98.83%, respectively, as shown in Table 20. The accuracy of the multi-class classification of each model could reach 98%. Table 20 lists the inference time required to execute each output. The enhancement in multi-class classification accuracy is mostly in the range of 0.01–0.05% for the combinations of CNN + RNN and CNN + LSTM compared to the individual DNN, RNN, CNN and LSTM methods. In addition, the inference time for the combinations of CNN + RNN and CNN + LSTM is longer than that of the individual DNN, RNN and CNN. When considering the implementation of the algorithm in the IDS device, since the IDS is installed in the data center, the data processing speed is high, so the inference time needs to be short. For this reason, the authors of [33] proposed a DNN-based network intrusion detection system which can detect cyber attack in real time in an IoT network. Therefore, the combination of CNN + RNN and CNN+ LSTM techniques is not encouraging compared to the individual techniques. Table 20 lists the accuracy and inference time of the six models. When considering that the deep learning model is implemented in the actual IDS device, the user can choose the best model that meets the inference time requirements.

As shown in Table 21, Table 22, Table 23, Table 24, Table 25 and Table 26, the DNN showed good results in the evaluation metrics of the benign samples and the six attack categories. With Infiltration, due to the small number of samples, the model could not be effectively analyzed in the learning stage. The precision, recall and F1-score evaluation metrics of the DNN model for the Infiltration category were all 0%. The precision, recall and F1-score evaluation metrics of the CNN model for the Infiltration category were 52.23%, 2.48% and 4.73%, respectively. It can be seen that with the small number of samples, the CNN could identify attacks better than the DNN. The precision, recall and F1-score of the RNN model for Infiltration were 47.06%, 2.11% and 3.99%, respectively. The LSTM model was better than the RNN model, and its recall and F1-score were slightly increased by 1.47% and 1.29%, respectively. However, the CNN is better than the LSTM and RNN models. The CNN + RNN and CNN models showed the same results in the Infiltration category. CNN + LSTM was the best of all models at identifying the Infiltration category, and its recall and F1-score increased by 0.73% and 0.58%, respectively.

We analyzed the BruteForce and Web Attack categories for a few samples. All the models showed poor results for Web Attack. The precision, recall and F1-score evaluation metrics of the DNN model for Web Attack were 100%, 37.50% and 54.55%, respectively, which were still not as good as those of the CNN, but better than those of the RNN and LSTM. The precision of CNN + RNN in the BruteForce category was 100%, which was the highest among all methods. 

Regarding DoS, Botnet and DDoS (which are commonly used by hackers today), this experiment showed that all obtained good results. The precision, recall and F1-score of the DoS category were all as high as 98~99%. In the Botnet attack category, the DNN, CNN, RNN and LSTM models all achieved a recall of more than 99%. The precision of CNN + RNN was 100%. DDoS had a large number of samples, but the obtained results were worse than those for DoS and Botnet, with an average of 98% for each method. The precision, recall and F1-score of the six models were all 98%. CNN + LSTM was the best of all the studied models.

Table 27 summarize the best results of each model for multi-class classification. In addition to integrating the four evaluation metrics, the training parameters and inference time of each model are also attached. DNN and CNN achieved a high accuracy in the deep network. The RNN needed only one shallow layer to reach an accuracy of 98.80% in multi-class classification, and the inference time was also shorter. The LSTM model could reach a 98.83% classification accuracy under one shallow layer. In this experiment, the characteristics of CNN feature extraction were applied to time-series RNN and LSTM models. In multi-class classification, both showed an accuracy of 98.84%, thus improving by 0.04% and 0.01%, respectively.

Most of the classification tasks tested in the literature are related to multi-class classification, and due to the huge amount of data in our studied dataset, there is no special quantitative standard for training and testing datasets. Table 28 lists a comprehensive comparison of our experimental results with related literature based on the CSE-CIC-IDS2018 dataset. Regarding the accuracy index, the DNN in this paper showed a value 1.55% higher than that reported in the literature [5] in multi-class and binary classification. Compared with the CNN methods of [10] and [5], our accuracy was 7.32% and 1.44% higher, respectively. Compared with the RNN method of [5], our accuracy was 1.51% higher. Compared with the method of TCN + LSTM [17], our accuracy was 2.64% and 1.06% higher, respectively. The accuracy results of the CNN + RNN and CNN + LSTM models of our experiment were also significantly higher than the aforementioned papers. Although the accuracy of [12] is 99.7% better than our paper, the paper does not use all datasets for training and testing, so it cannot be compared with our paper. Compared with [5,6,9,10,17], the proposed model effectively improves the detection performance.

#### 4.2.2. Evaluation of Binary Classification

Table 29 summarize the best results of each model for binary classification. In addition to integrating the four evaluation indicators, the training parameters and inference time of each model are also attached. The inference time of multi-class classification was longer than that of binary classification, because the number of classifications of attack samples in the output layer was different, so the judgment required more time in the processing and analysis of all attack data. DNN achieved a high accuracy in the deep network. The CNN achieved the highest accuracy under the same structure (CNN) for binary classifications. The RNN needed up to five layers to reach an accuracy of 98.82% in binary classification and the inference time was also shorter. The LSTM model could reach a 98.83% classification accuracy under similar layers. In this experiment, the characteristics of CNN feature extraction were applied to time-series RNN and LSTM models. In binary classification, CNN + RNN and CNN + LSTM demonstrated accuracy levels of 98.84% and 98.85%, respectively, which were both improved by 0.02% compared with the RNN and LSTM models. 

## 5. Conclusions

In this study, after data preprocessing using data cleaning, data transformation and split, and numerical normalization, the DNN, CNN, RNN, LSTM, CNN + RNN and CNN + LSTM models were used for the binary and multi-class classification of the CSE-CIC-IDS2018 dataset, and the accuracy of all models was found to reach more than 98%. The multi-class classification obtained the highest accuracy of 98.84% by the CNN + RNN and CNN + LSTM models. Compared with the IDS of other papers, the proposed model effectively improves the detection performance. 

There are minimal accuracy improvements at the cost of very high inference time for the combinations of CNN + RNN and CNN + LSTM compared to the individual DNN, RNN, CNN and LSTM methods. When considering the implementation issue in an IDS device, a shorter inference time of the deep learning structure is preferred. Because the accuracy of individual DNN, RNN, CNN and LSTM was found to reach more than 98%, they are more suitable than CNN + RNN and CNN + LSTM to realize the IDS device. In the future, we will study the feasibility of lightweight DNN, RNN, CNN and LSTM.

## Figures and Tables

**Table 1:** Summary of recent approaches.

Paper	Year	Dataset	Methods	Classification	Accuracy
[9]	2019	CSE-CIC-IDS2018	LSTM + AM	Multi-class	96.19%
[10]	2019	NSLKDD	Improved CNN	Multi-class	95.36%
[11]	2019	CSE-CIC-IDS2018	LSTM	Multi-class	96%
[12]	2020	CSE-CIC-IDS2018	Adaboost	Multi-class	99.69%
[5]	2020	CSE-CIC-IDS2018	DNN, RNN, CNN	Multi-class	97.28%, 97.31%, 97.38%
[13]	2020	NSLKDD	ADASYN + CNN	Multi-class	80.08%
[14]	2020	NSL-KDD UNSW-NB15	CNN + BiLSTM	Multi-class	83.58% 77.16%
[15]	2020	KDD99	LSTM	Binary	98.94%
[16]	2020	CSE-CIC-IDS2017	LSTM + CNN	Multi-class	98.60%
[17]	2021	KDD99 CSE-CIC-IDS2018	TCN + LSTM	Multi-class	92.05% 97.77%
[6]	2021	KDD99 CSE-CIC-IDS2018	TCN + LSTM	Multi-class	92% 97%
[18]	2021	NSLKDD	BiLSTM	Binary Multi-class	94.26% 91.36%
[19]	2021	KDD99	LSTM	Binary	98.88%
[20]	2022	Collected data	CNN + LSTM	Multi-class	97.30%
[21]	2022	NSLKDD	DSN	Multi-class	86.80%

**Table 2:** List of attack types.

Attack	Attack Name
Bruteforce	FTP-Bruteforce and SSH-Bruteforce
DoS	DoS-GoldenEye, DoS-Slowloris, DoS-SlowHTTPTest, and DoS-Hulk
Web Attack	Brute Force-Web, Brute Force-XSS, and SQL Injection
Infiltration	Infiltration
Botnet	Bot
DDoS	DDoS attacks-LOIC-HTTP, DDoS-LOIC-UDP, and DDOS-HOIC

**Table 3:** Original data, deleted non-attack data and data after cleaning.

Original Data	Remove Non Attack Data	Remove Features	Outlier Padding	Remove Duplicate Data	Processed Data
16,233,002 rows × 84 columns	59 rows	14 columns	—	5,369,992 rows	10,862,951 rows × 70 columns
	16,232,943 rows × 84 columns	16,232,943 rows × 70 columns	16,232,943 rows × 70 columns	10,862,951 rows × 70 columns	

The symbol (—) means no deleted data.

**Table 4:** CSE-CIC-IDS2018 binary classification of training-validation and testing data.

Label	Training-Validation Set		Testing Set	
	Number	Proportion	Number	Proportion
Benign	7,003,032	87.22%	1,824,935	87.49%
Attacks	1,025,894	12.78%	260,892	12.51%
total	8,028,926	100%	2,085,827	100%

**Table 5:** CSE-CIC-IDS2018 multi-class classification of training-validation and testing data.

Label	Training-Validation Set		Testing Set	
	Number	Proportion	Number	Proportion
Benign	7,003,032	87.22%	1,824,935	87.49%
DDoS	618,384	7.70%	154,529	7.41%
DoS	156,525	1.95%	39,443	1.89%
Botnet	114,647	1.43%	28,786	1.38%
Bruteforce	75,434	0.94%	18,619	0.89%
Infiltration	60,382	0.75%	19,379	0.93%
Web Attack	522	0.01%	136	0.01%
total	8,028,926	100%	2,085,827	100%

**Table 6:** Benchmarks of neural network architectures.

Hidden Layers	Total Number of Neurons	Units
1	256, 512, 768	256, 512, 768
2	256, 512, 768	256 (128 + 128), 512 (256 + 256), 768 (256 + 512)
3	256, 512, 768	256 (64 + 64 + 128), 512 (128 + 128 + 256), 768 (256 + 256 + 256)
4	256, 512, 768	256 (64 + 64 + 64 + 64), 512 (128 + 128 + 128 + 128), 768 (128 + 128 + 256 + 256)
5	256, 512, 768	256 (32 + 32 + 64 + 64 + 64), 512 (64 + 64 + 128 + 128 + 128), 768 (128 + 128 + 128 + 128 + 256)

**Table 7:** DNN-5-256 (5 layers and 256 neurons).

Layers	Type	Output Shape	Number of Units	Activation Function	Parameters
0~1	Dense	(None, 1, 64)	64		4480
1~2	BN	(None, 1, 64)			256
2~3	Dropout	(None, 1, 64)			0
3~4	Dense	(None, 1, 64)	64		4160
4~5	BN	(None, 1, 64)			256
5~6	Dropout	(None, 1, 64)			0
6~7	Dense	(None, 1, 64)	64		4160
7~8	BN	(None, 1, 64)			256
8~9	Dropout	(None, 1, 64)			0
9~10	Dense	(None, 1, 32)	32		2080
10~11	BN	(None, 1, 32)			128
11~12	Dropout	(None, 1, 32)			0
12~13	Dense	(None, 1, 32)	32		1056
13~14	BN	(None, 1, 32)			128
14~15	Dropout	(None, 1, 32)			0
15~16	Flatten	(None, 32)			0
16~17	Dense	(None, 1) (None, 7)	Binary 1 Multi 7	Sigmoid SoftMax	33 231

Total parameters: 16,993 (Binary); 17,191 (Multi-class). Trainable parameters: 16,481 (Binary); 16,679 (Multi-class). Non-trainable parameters: 512 (Binary); 512 (Multi-class).

**Table 8:** The number of parameters of the DNN model.

	Nodes	1 Layer	2 Layers	3 Layers	4 Layers	5 Layers
Binary	256	18,689	26,113	21,953	17,537	16,481
	512	37,377	84,993	68,481	59,649	55,489
	768	56,065	168,961	1,512,97	134,785	102,017
Multi-class	256	20,231	26,887	22,343	17,927	16,679
	512	40,455	86,535	69,255	60,423	5,5879
	768	60,679	170,503	152,839	135,559	102,791

**Table 9:** CNN-5-256 (5 layers and 256 neurons).

Layers	Type	Output Shape	Number of Filters	Kernel Size	Activation Function	Parameters
0~1	Conv1D	(None, 68, 32)	32	2 × 1	ReLU	96
1~2	MaxPooling	(None, 34, 32)				0
2~3	Conv1D	(None, 33, 32)	32	2 × 1	ReLU	2080
3~4	MaxPooling	(None, 16, 32)				0
4~5	Conv1D	(None, 15, 64)	64	2 × 1	ReLU	4160
5~6	MaxPooling	(None, 7, 64)				0
6~7	Conv1D	(None, 6, 64)	64	2 × 1	ReLU	8256
7~8	MaxPooling	(None, 3, 64)				0
8~9	Conv1D	(None, 2, 64)	64	2 × 1	ReLU	8256
9~10	MaxPooling	(None, 1, 64)				0
10~11	BN	(None, 1, 64)				256
11~12	Dropout	(None, 1, 64)				0
12~13	Flatten	(None, 64)				0
13~14	Dense	(None, 1) (None, 7)	Binary 1 Multi 7		Sigmoid SoftMax	65 455

Total parameters: 23,169 (Binary); 23,559 (Multi-class). Trainable parameters: 23,041 (Binary); 23,431 (Multi-class). Non-trainable parameters: 128 (Binary); 128 (Multi-class).

**Table 10:** The number of parameters of the CNN model.

	Nodes	1 Layer	2 Layers	3 Layers	4 Layers	5 Layers
Binary	256	9985	51,969	34,305	29,313	23,041
	512	19,969	202,241	134,145	148,737	91,137
	768	29,953	403,713	396,801	346,113	165,633
Multi-class	256	62,215	64,263	39,687	30,087	23,431
	512	124,423	226,823	144,903	150,279	91,911
	768	186,631	452,871	407,559	349,191	167,175

**Table 11:** RNN-5-256 (5 layers and 256 neurons).

Layers	Type	Output Shape	Number of Units	Activation Function	Parameters
0~1	SimpleRNN	(None, 1, 64)	64		8576
1~2	BN	(None, 1, 64)			256
2~3	Dropout	(None, 1, 64)			0
3~4	SimpleRNN	(None, 1, 64)	64		8256
4~5	BN	(None, 1, 64)			256
5~6	Dropout	(None, 1, 64)			0
6~7	SimpleRNN	(None, 1, 64)	64		8256
7~8	BN	(None, 1, 64)			256
8~9	Dropout	(None, 1, 64)			0
9~10	SimpleRNN	(None, 1, 32)	32		3104
10~11	BN	(None, 1, 32)			128
11~12	Dropout	(None, 1, 32)			0
12~13	SimpleRNN	(None, 32)	32		2080
13~14	BN	(None, 32)			128
14~15	Dropout	(None, 32)			0
15~16	Dense	(None, 1) (None, 7)	Binary 1 Multi 7	Sigmoid SoftMax	33 231

Total parameters: 31,329 (Binary); 31,527 (Multi-class). Trainable parameters: 30,817 (Binary); 31,015 (Multi-class). Non-trainable parameters: 512 (Binary); 512 (Multi-class).

**Table 12:** The number of parameters of the RNN model.

	Nodes	1 Layer	2 Layers	3 Layers	4 Layers	5 Layers
Binary	256	84,255	58,881	46,529	33,921	30,817
	512	299,521	216,065	166,785	125,185	112,833
	768	645,889	496,641	347,905	298,625	233,089
Multi-class	256	85,767	59,655	46,919	34,311	31,015
	512	302,599	217,607	167,559	125,959	113,223
	768	650,503	498,183	349,447	299,399	233,863

**Table 13:** LSTM-5-256 (5 layers and 256 neurons).

Layers	Type	Output Shape	Number of Units	Activation Function	Parameters
0~1	LSTM	(None, 1, 64)	64		34,304
1~2	BN	(None, 1, 64)			256
2~3	Dropout	(None, 1, 64)			0
3~4	LSTM	(None, 1, 64)	64		33,024
4~5	BN	(None, 1, 64)			256
5~6	Dropout	(None, 1, 64)			0
6~7	LSTM	(None, 1, 64)	64		33,024
7~8	BN	(None, 1, 64)			256
8~9	Dropout	(None, 1, 64)			0
9~10	LSTM	(None, 1, 32)	32		12,416
10~11	BN	(None, 1, 32)			128
11~12	Dropout	(None, 1, 32)			0
12~13	LSTM	(None, 32)	32		8320
13~14	BN	(None, 32)			128
14~15	Dropout	(None, 32)			0
15~16	Dense	(None, 1) (None, 7)	Binary 1 Multi 7	Sigmoid SoftMax	33 231

Total parameters: 122,145 (Binary); 122,343 (Multi-class). Trainable parameters: 121,633 (Binary); 121,831 (Multi-class). Non-trainable parameters: 512 (Binary); 512 (Multi-class).

**Table 14:** The number of parameters of the LSTM model.

	Nodes	1 Layer	2 Layers	3 Layers	4 Layers	5 Layers
Binary	256	334,593	233,601	184,385	133,953	121,633
	512	1,193,473	860,417	663,681	497,281	448,065
	768	2,576,641	1,981,185	1,386,241	1,189,505	927,361
Multi-class	256	336,135	234,375	184,775	134,343	121,831
	512	1,196,551	861,959	664,455	498,055	448,455
	768	2,581,255	1,982,727	1,387,783	1,190,279	928,135

**Table 15:** CNN + RNN-8-256 (8 layers and 256 neurons).

Layers	Type	Output Shape	Number of Units	Kernel Size	Activation Function	Parameters
0~1	Conv1D	(None, 68, 32)	32	2*1	ReLU	96
1~2	MaxPooling	(None, 34, 32)				0
2~3	Conv1D	(None, 32, 64)	64	3*1	ReLU	6208
3~4	MaxPooling	(None, 16, 64)				0
4~5	Conv1D	(None, 14, 128)	128	3*1	ReLU	24,704
5~6	MaxPooling	(None, 7, 128)				0
6~7	BN	(None, 7, 128)				512
7~8	Dropout	(None, 7, 128)				0
8~9	SimpleRNN	(None, 7, 64)	64			12,352
9~10	BN	(None, 7, 64)				256
10~11	Dropout	(None, 7, 64)				0
11~12	SimpleRNN	(None, 7, 64)	64			8256
12~13	BN	(None, 7, 64)				256
13~14	Dropout	(None, 7, 64)				0
14~15	SimpleRNN	(None, 7, 64)	64			8256
15~16	BN	(None, 7, 64)				256
16~17	Dropout	(None, 7, 64)				0
17~18	SimpleRNN	(None, 7, 32)	32			3104
18~19	BN	(None, 7, 32)				128
19~20	Dropout	(None, 7, 32)				0
20~21	SimpleRNN	(None, 32)	32			2080
21~22	BN	(None, 32)				128
22~23	Dropout	(None, 32)				0
23~24	Dense	(None, 1) (None, 7)	Binary 1 Multi 7		Sigmoid SoftMax	33 231

Total parameters: 66,625 (Binary); 66,823 (Multi-class). Trainable parameters: 65,857 (Binary); 66,055 (Multi-class). Non-trainable parameters: 768 (Binary); 768 (Multi-class).

**Table 16:** The number of parameters of the CNN + RNN model.

	Nodes	1 Layer	2 Layers	3 Layers	4 Layers	5 Layers
Binary	256	130,593	97,697	85,345	68,961	65,857
	512	360,993	262,433	213,153	164,001	151,649
	768	722,465	558,113	394,273	344,993	279,457
Multi-class	256	132,135	98,471	85,735	69,351	66,055
	512	364,071	263,975	213,927	164,775	152,039
	768	727,079	559,655	395,815	345,767	2802

**Table 17:** CNN + LSTM-8-256 (8 layers and 256 neurons).

Layers	Type	Output Shape	Number of Units	Kernel Size	Activation Function	Parameters
0~1	Conv1D	(None, 68, 32)	32	2*1	ReLU	96
1~2	MaxPooling	(None, 34, 32)				0
2~3	Conv1D	(None, 32, 64)	64	3*1	ReLU	6208
3~4	MaxPooling	(None, 16, 64)				0
4~5	Conv1D	(None, 14, 128)	128	3*1	ReLU	24,704
5~6	MaxPooling	(None, 7, 128)				0
6~7	BN	(None, 7, 128)				512
7~8	Dropout	(None, 7, 128)				0
8~9	LSTM	(None, 7, 64)	64			49,408
9~10	BN	(None, 7, 64)				256
10~11	Dropout	(None, 7, 64)				0
11~12	LSTM	(None, 7, 64)	64			33,024
12~13	BN	(None, 7, 64)				256
13~14	Dropout	(None, 7, 64)				0
14~15	LSTM	(None, 7, 64)	64			33,024
15~16	BN	(None, 7, 64)				256
16~17	Dropout	(None, 7, 64)				0
17~18	LSTM	(None, 7, 32)	32			12,416
18~19	BN	(None, 7, 32)				128
19~20	Dropout	(None, 7, 32)				0
20~21	LSTM	(None, 32)	32			8320
21~22	BN	(None, 32)				128
22~23	Dropout	(None, 32)				0
23~24	Dense	(None, 1) (None, 7)	Binary 1 Multi 7		Sigmoid SoftMax	33 231

Total parameters: 168,769 (Binary); 168,967 (Multi-class). Trainable parameters: 168,001 (Binary); 168,199 (Multi-class). Non-trainable parameters: 768 (Binary); 768 (Multi-class).

**Table 18:** The number of parameters of the CNN + LSTM model.

	Nodes	1 Layer	2 Layers	3 Layers	4 Layers	5 Layers
Binary	256	426,273	295,073	245,857	180,321	167,937
	512	1,345,569	952,097	755,361	558,753	509,537
	768	2,789,153	1,673,633	1,477,921	1,281,185	1,019,041
Multi-class	256	427,815	295,847	246,247	180,711	168,199
	512	1,348,647	953,639	756,135	559,527	509,927
	768	2,793,767	2,134,823	1,479,463	1,281,959	1,019,815

**Table 19:** Experimental environment.

Project	Properties
OS	Ubuntu 18.04.4 LTS
CPU	Intel(R) Xeon(R) CPU E5-2698 v4 @ 2.20GHz
GPU	NVIDIA Tesla V100-SXM2-32GB-LS
Memory	62.88GiB
Disk	7.0TiB
Python	Python 3.6.9
NVIDIA CUDA	11.0
Framework	TensorFlow 2.2.0+nv- tf2-py3

**Table 20:** The accuracy and inference time of DNN, CNN, RNN, LSTM, CNN + RNN and CNN + LSTM for multi-class classification.

	No. of Node	1 Layer		2 Layer		3 Layer		4 Layer		5 Layer	
		Acc	Infer Time (ms)	Acc	Infer Time (ms)	Acc	Infer Time (ms)	Acc	Infer Time (ms)	Acc	Infer Time (ms)
DNN	256	98.78%	2.15	98.80%	2.45	98.80%	2.78	98.78%	3.10	98.79%	3.42
	512	98.79%	2.18	98.80%	2.50	98.82%	2.79	98.82%	3.13	98.78%	3.39
	768	98.60%	2.15	98.82%	2.53	98.79%	2.88	98.79%	3.16	98.83%	3.42
CNN	256	98.53%	1.89	98.62%	2.18	98.79%	2.18	98.58%	2.36	98.79%	2.49
	512	98.56%	2.01	98.63%	2.41	98.79%	2.24	98.80%	2.45	98.81%	2.49
	768	98.57%	2.18	98.64%	2.49	98.81%	2.65	98.83%	2.64	98.81%	2.52
RNN	256	98.78%	2.09	98.78%	2.32	98.78%	2.34	98.77%	2.81	98.67%	3.05
	512	98.78%	2.03	98.79%	2.38	98.79%	2.36	98.78%	2.82	98.78%	3.10
	768	98.80%	2.09	98.75%	2.38	98.79%	2.72	98.79%	2.95	98.79%	3.13
LSTM	256	98.81%	2.12	98.79%	2.68	98.79%	2.98	98.83%	3.45	98.79%	3.94
	512	98.80%	2.21	98.83%	2.62	98.83%	3.02	98.83%	3.51	98.83%	3.87
	768	98.80%	2.18	98.83%	2.58	98.79%	3.05	98.83%	3.59	98.82%	4.02
CNN + RNN	256	98.80%	3.16	98.83%	3.77	98.83%	4.71	98.82%	5.55	98.83%	5.95
	512	98.84%	3.27	98.79%	4.17	98.83%	4.89	98.83%	5.23	98.83%	6.00
	768	98.84%	3.39	98.84%	4.49	98.82%	5.43	98.84%	5.15	98.83%	8.01
CNN + LSTM	256	98.83%	3.68	98.80%	4.50	98.82%	5.60	98.82%	5.51	98.79%	6.72
	512	98.83%	4.11	98.80%	4.37	98.83%	5.43	98.83%	6.31	98.80%	7.03
	768	98.84%	4.31	98.80%	4.74	98.84%	5.23	98.79%	6.49	98.84%	7.52

Acc: accuracy. Infer Time: inference time.

**Table 21:** DNN evaluation results for multi-class classification.

Class	Precision	Recall	F1-Score
Benign	98.80%	99.88%	99.33%
BruteForce	99.97%	99.92%	99.95%
DoS	99.06%	98.77%	98.91%
Web Attack	100%	37.50%	54.55%
Infiltration	0%	0%	0%
Botnet	99.98%	99.62%	99.80%
DDoS	98.76%	98.62%	98.69%

**Table 22:** CNN evaluation results for multi-class classification.

Class	Precision	Recall	F1-Score
Benign	98.83%	99.85%	99.34%
BruteForce	99.98%	99.94%	99.96%
DoS	99.08%	98.78%	98.93%
Web Attack	100%	58.09%	73.49%
Infiltration	52.23%	2.48%	4.73%
Botnet	99.98%	99.74%	99.86%
DDoS	98.68%	98.72%	98.70%

**Table 23:** RNN evaluation results for multi-class classification.

Class	Precision	Recall	F1-Score
Benign	98.82%	99.83%	99.32%
BruteForce	99.95%	99.91%	99.93%
DoS	98.77%	98.63%	98.70%
Web Attack	100%	36.03%	52.97%
Infiltration	47.06%	0.37%	0.74%
Botnet	99.86%	99.47%	99.67%
DDoS	98.30%	98.90%	98.60%

**Table 24:** LSTM evaluation results for multi-class classification.

Class	Precision	Recall	F1-Score
Benign	98.80%	99.87%	99.33%
BruteForce	99.92%	99.94%	99.93%
DoS	99.04%	98.75%	98.89%
Web Attack	98.08%	37.50%	54.26%
Infiltration	46.15%	0.19%	0.37%
Botnet	99.96%	99.50%	99.73%
DDoS	98.78%	98.63%	98.71%

**Table 25:** CNN + RNN evaluation results for multi-class classification.

Class	Precision	Recall	F1-Score
Benign	98.86%	99.83%	99.34%
BruteForce	100%	99.91%	99.95%
DoS	99.12%	98.75%	98.93%
Web Attack	100%	58.09%	73.49%
Infiltration	53.25%	2.41%	4.61%
Botnet	100%	99.75%	99.87%
DDoS	98.42%	99.02%	98.72%

**Table 26:** CNN + LSTM evaluation results for multi-class classification.

Class	Precision	Recall	F1-Score
Benign	98.85%	99.84%	99.34%
BruteForce	99.99%	99.93%	99.96%
DoS	99.06%	98.78%	98.92%
Web Attack	100%	58.82%	74.07%
Infiltration	52.01%	3.07%	5.08%
Botnet	99.99%	99.75%	99.87%
DDoS	98.70%	98.83%	98.77%

**Table 27:** Comparison results of multi-class classification.

Methods	Accuracy	Precision	Recall	F1-Score	Trainable Parameters	Inference Time
DNN	98.83%	97.91%	98.83%	98.36%	20,231	3.421 ms
CNN	98.83%	98.42%	98.83%	98.41%	349,191	2.638 ms
RNN	98.80%	98.33%	98.80%	98.35%	650,503	2.086 ms
LSTM	98.83%	98.34%	98.83%	98.37%	134,343	3.451 ms
CNN + RNN	98.84%	98.43%	98.84%	98.42%	364,071	3.068 ms
CNN + LSTM	98.84%	98.43%	98.84%	98.43%	2,793,767	4.31 ms

**Table 28:** Comparison with related literature based on the CSE-CIC-IDS2018 dataset.

Paper	Year	Methods	Accuracy	Our Methods	Accuracy
[10]	2019	CNN	91.5%		
[9]	2019	LSTM + AM	96.19%		
[5]	2020	DNN RNN CNN	97.28% 97.31% 97.38%	DNN RNN CNN	98.83% 98.83% 98.80%
[12]	2020	AdaBoost	99.69%	LSTM	98.83%
[17]	2020	TCN + LSTM	97.77%	CNN + RNN	98.84%
[6]	2021	TCN + LSTM	97%	CNN + LSTM	98.84%

**Table 29:** Comparison results of binary classification.

Methods	Accuracy	Precision	Recall	F1-Score	Trainable Parameters	Inference Time
DNN	98.83%	98.83%	98.83%	98.81%	18,689	3.359 ms
CNN	98.82%	98.82%	98.82%	98.80%	346,113	2.301 ms
RNN	98.82%	98.82%	98.82%	98.81%	233,089	2.807 ms
LSTM	98.83%	98.83%	98.83%	98.81%	927,361	4.019 ms
CNN + RNN	98.84%	98.84%	98.84%	98.82	85,345	4.341 ms
CNN + LSTM	98.85%	98.85%	98.85%	98.83%	1,345,569	3.068 ms

## Data Availability

The data are available upon request.
